# Integrative proteomic and physiological analyses of the molecular response to dessication-stress in *Auricularia fibrillifera*

**DOI:** 10.3389/fpls.2022.995810

**Published:** 2022-09-21

**Authors:** Hao Guo, Xingwei Xiong, Yiqin Wang, Huaizhi Tian, Suqin Zhang, Guangdong Geng

**Affiliations:** College of Agriculture, Guizhou University, Guiyang, Guizhou, China

**Keywords:** *Auricularia fibrillifera*, proteome, dessication stress, melanin, antibiotics, folate, biotin, cytoskeleton

## Abstract

Drought stress is one of the main factors influencing the growth and development of an organism. *Auricularia fibrillifera* has strong dessication resistance. In *A. fibrillifera* under dessication-stress, the melanin content of fruiting bodies elevated significantly by >10-fold compared with the control. Folate content also increased sharply but decreased significantly after rehydration, and amino acid and biotin levels increased by 40.11 and 22.14%, respectively. In proteomic analysis, 1,572 and 21 differentially abundant proteins (DAPs) were identified under dessication-stress and rehydration, respectively. A large number of DAPs were annotated in “amino acid metabolism,” “carbohydrate metabolism,” and “translation” pathways, and the DAPs related to osmotic regulation and antioxidant enzymes were significantly increased in abundance. Transcriptome-proteome association analysis showed that most DAPs (30) were annotated in the “biosynthesis of antibiotics” pathway. DAPs and corresponding differentially expressed genes were all up-regulated in the “biotin biosynthesis” pathway and associated with “folate biosynthesis” and “phenylalanine, tyrosine, and tryptophan biosynthesis.” In the analysis of protein–protein interactions, the DAPs annotated in the “phenylalanine, tyrosine, and tryptophan biosynthesis” pathway had the strongest interactions with other DAPs. These enriched pathways could enhance amino acid, folate, biotin, and melanin levels during desiccation stress, which is consistent with the physiological data (amino acid, folate, biotin, and melanin contents). In addition, many DAPs related to the cytoskeleton were significantly increased in abundance under dessication-stress. Physiological and transcriptome data were in agreement with proteomic results. This work provides valuable insight into the dessication-tolerant mechanisms of *A. fibrillifera.*

## Introduction

*Auricularia fibrillifera* is a common traditional Chinese food and medicine, which is the third most important cultivated mushroom worldwide (Yuan et al., 2019). It has a pleasant taste and many health-associated characteristics such as antioxidant, anticoagulant, antitumor, immunomodulatory, and cholesterol-lowering properties (Sekara et al., 2015). It is expected that the duration and severity of droughts will increase, resulting in adverse effects on agriculture and causing significant declines in crop production on a global scale (Lesk et al., 2016). Thus, it is important to improve drought tolerance in organisms for global food security and necessary to clarify the physiological and molecular mechanisms of dessication tolerance.

Some mechanisms of drought response are similar between *A. fibrillifera* and plant species (Wang et al., 2016, 2022; Zandalinas et al., 2018; Mahmood et al., 2019). The physiological effects induced by drought stress include altered cell wall elasticity, increased oxidative stress (Caruso et al., 2009), toxic metabolite generation, and extensive cellular damage in plants (Ahuja et al., 2010; Zandalinas et al., 2018). Plants have also evolved various drought tolerance mechanisms to adapt to drought stress. The drought resistance of plants is quite complex (Wang et al., 2015). Osmotic adjustment, hormonal regulation, antioxidant systems, and signal transduction play vital roles in drought tolerance (Zandalinas et al., 2018; Mahmood et al., 2019). Amino acids have crucial roles in osmotic adjustment. Melanins exist in fungi, plants, and microorganisms, and have the functions of free-radical scavenging, antioxidant activity, and radiation protection (Burmasova et al., 2019; Cordero and Casadevall, 2020; Cao et al., 2021). Biotin and biotinylation might be involved in energy management to cope with drought and flooding in the early stage of soybean-root tip (Wang et al., 2016). Foliar application of folate was found to be not only suitable for drought stress alleviation in *Coriandrum sativum* L. but also beneficial for improvement in growth and yield under water-deficit circumstances (Khan et al., 2022).

A loss of more than 10% of plant fresh weight can lead to water stress, which can induce the synthesis of some specific ones (including S-like RNase homolog, actin depolymerizing factor, rubisco activase, and translational initiation factor EF-Tu), maintain others, and decrease the levels of some plant proteins (such as isoflavone reductase-like protein and chloroplast Rieske Fe-S protein) (Hsiao, 1973; Salekdeh et al., 2002). Investigating the proteome profiles under drought stress can provide detailed information regarding the specific protein changes associated with drought responses (Koh et al., 2015). Under drought stress response/tolerance, numerous proteins related to metabolism, photosynthesis, stress, and defense were identified by a comparative proteome analysis in *Brassica napus* seedlings (Koh et al., 2015). Proteomic studies on post-drought recovery have clarified the mechanisms of plants in response to drought stress (Khan and Komatsu, 2016). Four novel drought-responsive proteins were identified during drought stress and recovery in rice leaves by proteomic analysis (Salekdeh et al., 2002). Aldehyde dehydrogenase and peroxidase are known to decrease aldehydes and toxic reactive oxygen species from soybean roots and help in the recovery from drought stress (Khan and Komatsu, 2016). Under drought stress, the abundance of most protein changes may be associated with gene transcription. There was a positive correlation between protein expression and gene transcription in *B. napus*, although different patterns between proteins and transcripts were detected at various time points (Koh et al., 2015). These reports provide valuable information for investigating the molecular mechanisms of dessication tolerance in *A. fibrillifera*.

For general organisms (the majority of terrestrial plants and mushrooms), the vegetative bodies will dry up or even die under severe drought stress. *Selaginella lepidophylla* is a desiccation-tolerant plant capable of surviving complete vegetative tissue dehydration and reviving under water conditions (Pampurova and Van Dijck, 2014). A candidate basic helix-loop-helix (bHLH) transcription factor was observed to be highly expressed at 4% relative water content in *S. lepidophylla* (*SlbHLH*), and its overexpression significantly increased integrated water-use efficiency and green cotyledon emergence rates under water-deficit stress in *Arabidopsis* (Ariyarathne and Wone, 2022). The fruit bodies of *Auricularia* dries out and enters dormancy under dessication conditions. The dormancy may be broken once watered. Hence, the dessication tolerance and rehydration capability of *A. fibrillifera* make it a suitable model to elucidate its adaptive mechanism against dessication-stress (Ma et al., 2014). Currently, there are limited reports on the molecular mechanisms of dessication tolerance in *A. fibrillifera*. In this study, protein markers and pathways were investigated under dessication-stress in *A*. *fibrillifera* by data-independent acquisition (DIA) proteomic profiling to explore the dessication-tolerant mechanism of *A*. *fibrillifera* and provide novel information for dessication-tolerant breeding for *A*. *fibrillifera*.

## Materials and methods

### Materials and treatments

A dessication-tolerant *A. fibrillifera* cultivar “CSLZ” was used for this study. The strain was maintained in a culture medium to generate fruiting bodies (Wang et al., 2022). Upon reaching full mycelial colonization, polyethylene bags were removed, and the substrate was cultured at 25 ± 1°C under a 15:9 h-light/dark cycle. The substrate-containing bags were routinely sprayed with 15 mL water/bag 8 times daily. When the diameters of the fruit bodies reached 2-3 cm, dessication-stress treatment was initiated, and the fruit bodies on the substrate naturally dehydrated. The regularly watered fruit bodies on the substrate served as a parallel control. When the water loss rate of fruit bodies achieved 60% (desiccation-stress, DS) compared to the CK1 parallel control, uniformly sized fruit bodies were harvested as the first samples. The fruit bodies were rewatered. The next sampling process was conducted when the water loss rate of fruit bodies was 50% [after rehydration (RE) for 1 h] compared to the CK2 parallel control. Each sample pool included 15 individual fruit bodies, and the experiment was performed in triplicate. All the specimens were immediately frozen in liquid nitrogen and kept at -80°C for further analysis.

### Physiological analysis

The amino acid (Cas No.: BC1575, Solarbio, Beijing, China), biotin (Cas No.: BC4804, Solarbio), and folate (Cas No.: BC4834, Solarbio) contents were determined according to the kit instructions. In brief, the α-amino group of amino acids can react with hydrated ninhydrin to produce a blue-violet compound with an absorption peak at 570 nm, which was detected with a microplate reader (Thermo-Fisher-Scientific, San Jose, CA, United States). Both biotin and folate have ultraviolet absorption at 210 nm, and their contents were determined by high performance liquid chromatography (HPLC) (Shimadzu, Kyoto, Japan) and ultraviolet detector (Shimadzu). Melanin was extracted using a method by Wang et al. (2022). Shortly, 1.0 g fruit body was ground in 50 mL of 1 M NaOH. The samples were treated in an ultrasonic cleaner (300 W) for 2 h at 60°C. The supernatant (pH adjusted to 1.5) was immersed in a boiling water bath for 10 h, and then centrifuged at 9,156 × *g* for 15 min. After air-drying the precipitation, the melanin content was calculated.

### Protein extraction

Total proteins of fruit bodies were isolated according to the phenol method (Isaacson et al., 2006) with slight modifications: briefly, 0.5 g fruit bodies were ground into a fine powder in a lysis buffer containing 877 mM sucrose, 100 mM EDTA, 20 mM Tris-HCL (pH = 8.0), 1 mM dithiothreitol (DTT), 2% (v:v) β-mercaptoethanol, 1% (v:v) Triton X-100, and 0.1 × Cocktail (Roche, Switzerland). Subsequently, 2 × volume of tris-saturated phenol (pH = 7.5) was added and centrifuged at 25,000 × *g* for 15 min at 4°C. After collecting the supernatant, 5 × volume of precooled precipitate solution, containing 0.1 M ammonium acetate in methanol and 10 mM DTT, was added to the protein mixture. Every sample was maintained for 2 h at -20°C. Then, the samples were centrifuged at 25,000 *g* for 15 min at 4°C, and the supernatant was removed. The pellets were further washed with 1 mL of precooled acetone [precooled acetone:sample = 5:1 (v/v)] with centrifugation at 25,000 × *g* for 15 min at 4°C. After air-drying the pellets, 200 μL of L3 lysis buffer containing 7 M urea, 2 M thiourea, 20 mM Tris, 10 mM DTT, and 1 × Cock-tail (Roche) were added, ground (60 Hz, 2 min), and centrifuged at 25,000 × *g* for 15 min at 4°C. DTT (10 mM) was added to the supernatant and it was kept in a water bath at 56°C for 1 h. Subsequently, iodoacetamide (55 mM) was added and kept in the dark for 45 min and, after adding 1 mL cold acetone, all samples were maintained 2 h at -20°C. All samples were centrifuged at 25,000 × *g* for 15 min at 4°C. After removing the supernatants, the pellets were air-dried and dissolved in 200 μL L3 lysis buffer. Trypsin [protein:trypsin = 40:1 (w/w)] was added for enzymolysis, and then desalinated, vacuum-dried and redissoluted. Nanodrop ND-1000 (Thermo-Fisher-Scientific) was used to measure protein concentrations.

### Liquid chromatography-tandem mass spectrometry analysis

As an internal standard for quantification, 2 mL of mixed extract solution (100 μg/mL) were used. After HPLC (Shimadzu) fractionation, the eluents were combined into 10 fractions for LC-tandem mass spectrometry (MS/MS). The freeze-dried peptides were dissolved in Solvent A (2% acetonitrile and 0.1% formic acid), and the peptide specimens were separated using an UltiMate 3000 UHPLC (Thermo-Fisher-Scientific). The analytical conditions were as follows: LC column, C18 (150 μm × 35 cm, 1.8 μm, 100 Å); gradient program, 5% B (98% acetonitrile and 0.1% formic acid) for 5.0 min, 5%–25% B for 115 min, 25%–35% B for 40 min, 35%–80% B for 10 min, 80% B for 5 min, and 5% B for 5 min; and flow rate, 300 nL/min. The peptide specimens were ionized by a nanoESI, and then put into a Q-Exactive HF tandem mass spectrometer (Thermo-Fisher-Scientific) for data-dependent acquisition mode detection (Tsou et al., 2015). The spectra of first-grade MS (MS1) were acquired in the scan range of 350–1,500 m/z with spray voltage of 1.6 kV, resolution of 120,000, automatic gain control (AGC) target of 3E^6^, and maximum injection time (MIT) of 50 ms. The spectra of second-grade MS (MS2) were obtained using the following parameters: resolution of 30,000, MIT of 100 ms, dynamic exclusion duration of 30 s, and AGC target of 1E^5^. Moreover, the mode of MS2 spectra was high-energy collisional dissociation (HCD), and the collision energy was 28%. For DIA analysis, the same nano-LC system and gradient were used as those employed for data-dependent acquisition analysis. The following were the DIA MS parameters: scan range of 400–1,250 m/z, resolution of 120,000, and MIT of 50 ms. The DIA isolation window was set to 17 m/z with loop count of 50, automatic MIT, resolution of 30,000, stepped collision energy of 22.5%, 25.0%, and 27.5%, and AGC target of 1E^6^.

Spectronaut was employed to efficiently deconvolute, precisely identify, and quantitatively analyze the data (Bernhardt et al., 2014).

### Identification of differentially abundant protein and bioinformatics analysis

The peak areas of an ion pair were extracted using Spectronaut (Bruderer et al., 2015). The error correction and normalization steps were performed using the Msstats software package (Choi et al., 2014). The DAPs fit these two criteria, fold-change ≥ 2 and Q-value ≤ 0.05. The consistency and probability of DAPs were scored and compared with the Swissprot library, and the threshold was set as 1E^–5^ for protein description. The subcellular localization, protein-protein interaction (PPI) and Kyoto Encyclopedia of Genes and Genomes (KEGG) enrichment analyses were performed with these DAPs. A hypergeometric test was used to detect significantly enriched pathways (*p* < 0.05).

### Proteome-transcriptome-associated analysis

We carried out proteome-transcriptome-associated analysis to gain a deeper understanding of the biological functions by combining results of two separate-omics techniques. The samples were analyzed at both mRNA and protein levels. The transcriptome data were retrieved from our previous study (NCBI SRA database under accession no. PRJNA695780) (Wang et al., 2022). Differentially expressed genes (DEGs) were chosen by DESeq software according to the following criteria: fold-change ≥ 2 and Q-value ≤ 0.001. Correlation analyses were performed between DAPs and DEGs of two omics, and the associated DAPs/DEGs were used for expression correlation analysis and metabolic pathway map integration analysis.

### Construction of protein-protein interaction network

STRING v11.5 (string-db.org) was applied to analyze the PPIs of DAPs identified in this study and their PPI network was constructed. The minimum required interaction score parameter was set at a high confidence level (0.70).

### Quantitative real-time polymerase chain reaction assays

Total RNA was isolated from fruiting bodies using an RNApre Pure Plant Plus Kit (Polyphenolics & Polysaccharides-rich) (Tiangen, Beijing, China). Using FastKing gDNA Dispelling RT SuperMix Kit (Tiangen), the extracted RNA was reverse-transcribed by following the manufacturer’s kit. Specific primers of 16 selected DAPs were designed using Primer 5 (Supplementary Table 1). The *18S* gene was used for reference (Zhao et al., 2019). The qRT-PCR amplification was conducted on an ABI StepOne Real-Time polymerase chain reaction (PCR) System (Applied Biosystems, CA, United States). The relative expression levels of target genes were determined using the 2^–ΔΔ^*^Ct^* method with 3 technical and biological replications (Livak and Schmittgen, 2001).

### Statistical analysis

Statistical tests were conducted with SPSS v19.0 (IBM Corp., NY, United States). The differences between means were compared with ANOVA followed by Duncan’s multiple range test. Pearson’s correlation analysis of binary variables was carried out. Level of statistical significance was set at *p* < 0.05.

## Results

### Physiological responses to dessication-stress and rehydration

**FIGURE 1:**
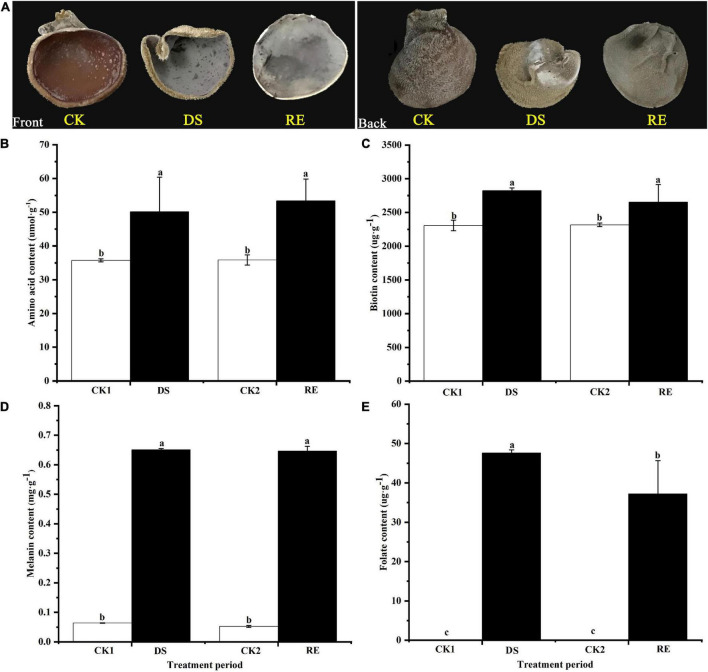
Phenotype and physiological responses to dessication-stress and rehydration in *Auricularia fibrillifera*. **(A)** Phenotypes of fruiting bodies. The front is on the left and the back is on the right. **(B)** Amino acid, **(C)** biotin, **(D)** melanin, and **(E)** folate content. CK1, CK2, DS, and RE represent the parallel control of desiccation-stress, parallel control of rehydration, desiccation-stress, and rehydration process, respectively. Bars indicate mean ± SD (*n* = 3). Values with different letters are significantly different at *p* < 0.05.

The phenotype of fruiting bodies changed significantly under dessication-stress. In response to dessication, the fruiting bodies shrank and became hard but after rehydration, they began to expand and appeared softened (Figure 1A). Levels of amino acids and biotin in fruiting bodies increased significantly by 40.11 and 22.14%, respectively, under dessication-stress compared with those of the controls (Figures 1B,C). The melanin content increased significantly by > 10-fold compared to the control (Figure 1D). The folate peak was not detected in the control, which might be owing to its extremely low level; but levels increased rapidly under dessication-stress. After rehydration, the decrease in folate was the greatest (21.79%) compared with amino acids, biotin, and melanin (Figure 1E).

### Differentially abundant proteins under dessication-stress

**FIGURE 2:**
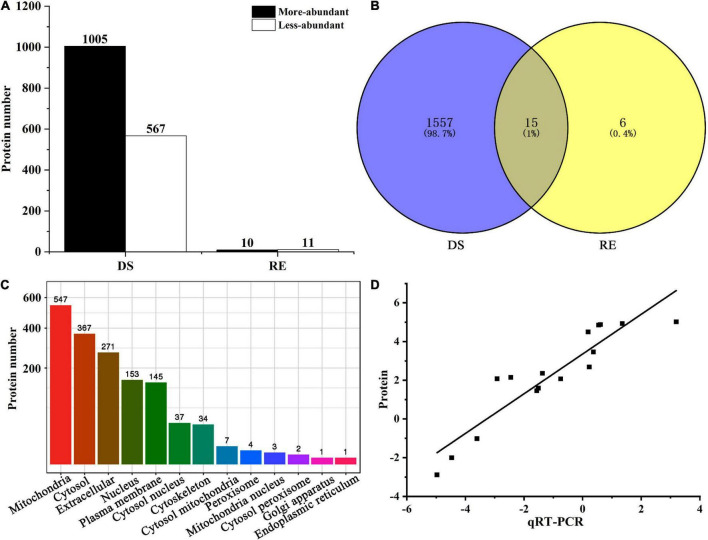
Number **(A)**, Venn diagram **(B)**, subcellular localization **(C)**, and correlation coefficient of differentially abundant proteins between proteome and qRT-PCR **(D)**. DS and RE represent dessication-stress and rehydration, respectively.

The proteomic analysis of fruiting bodies was performed during dessication-stress and rehydration stages, and stable results were obtained among the replicates of each treatment (Supplementary Figure 1). A total of 1,572 DAPs (1,005 more-abundant and 567 less-abundant) were observed under dessication-stress, and 10 more-abundant and 11 less-abundant DAPs were found after rehydration (Figure 2A and Supplementary Table 2). Fifteen DAPs appeared under both dessication-stress and rehydration (Figure 2B). The number of DAPs under dessication-stress was 74.86-fold of that after rehydration. Subcellular localization showed that under dessication-stress the DAPs were mainly located in mitochondria, followed by the cytoplasm and extracellular locations (Figure 2C).

To verify the reliability of proteome data, we selected 16 DAPs for qRT-PCR assays (Supplementary Table 3). The correlation coefficient of the fold-change values between proteome and qRT-PCR was statistically significant (*r* = 0.84, *p* = 3.80E^–7^; Figure 2D). The more- or less-abundant of the proteins, as revealed by proteomics analysis, was corroborated by qRT-PCR.

### Kyoto encyclopedia of genes and genomes enrichment analysis under dessication-stress

**FIGURE 3:**
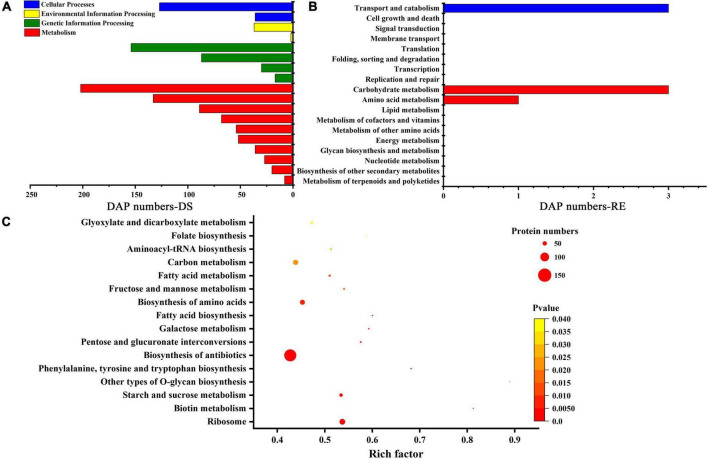
Kyoto Encyclopedia of Genes and Genomes (KEGG) pathways. **(A)** KEGG pathways under dessication-stress. **(B)** KEGG pathways after rehydration. **(C)** Significantly enriched pathways under dessication-stress. DS, RE, and DAP represent dessication-stress, rehydration, and differentially abundant protein, respectively.

In plants, different proteins coordinate with each other to activate cellular responses, and pathway analysis is helpful in further understanding biological function. Under dessication-stress, there were 874 DAPs annotated to different pathways, among these most DAPs were enriched in “metabolism” pathways, such as “carbohydrate metabolism” (202) and “amino acid metabolism” (133) (Figure 3A and Supplementary Table 4). Many DAPs were also annotated in “translation” (154) and “transport and catabolism” (127) pathways. After rehydration, the DAP number decreased rapidly, and only six DAPs were enriched in KEGG pathways (Figure 3B). Some DAPs were not annotated to any KEGG pathway, while some DAPs were in more than one KEGG pathway at desiccation and rehydration stages. “Glyoxylate and dicarboxylate metabolism,” “folate biosynthesis,” “fructose and mannose metabolism,” “biosynthesis of antibiotics,” “biosynthesis of amino acids,” “phenylalanine, tyrosine and tryptophan biosynthesis,” “biotin metabolism,” and other pathways were significantly enriched during the desiccation stress (Figure 3C). The highest number of DAPs occurred in the “biosynthesis of antibiotics” pathway (Figure 3C). There was no significantly enriched pathway after rehydration.

### Proteome-transcriptome-associated analysis

**FIGURE 4:**
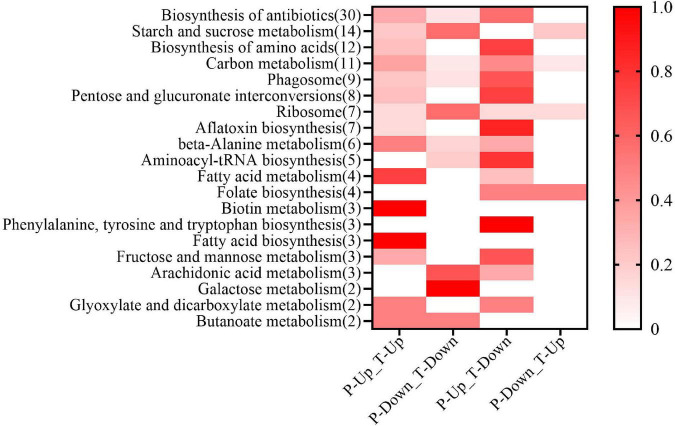
Proteome–transcriptome-associated Kyoto Encyclopedia of Genes and Genomes (KEGG) analysis. In the horizontal axis, T, P, Up, and Down show differentially expressed genes from the transcriptome, differentially abundant proteins from the proteome, up-regulation, and down-regulation, respectively. On the Y axis, the number of differentially abundant proteins in each pathway is shown in parentheses. The color of the heat map represents the proportion of proteins in the pathway.

The joint proteome and transcriptome analysis was useful in finding the regulation of gene expression (Maier et al., 2009). A total of 391 DAPs were associated with dessication-stress (Supplementary Tables 5, 6). The expression of corresponding DAPs and DEGs were focused mainly on two patterns: (1) both were up-regulated and (2) the DAPs were increased in abundance but the DEGs were down-regulated (Figure 4). The main associated pathways were “starch and sucrose metabolism,” “biosynthesis of antibiotics” and “biosynthesis of amino acids.” Interestingly, in the “biotin metabolism” pathway, all DAPs were associated, and both DAPs and DEGs were up-regulated. In the “folate biosynthesis,” “phenylalanine, tyrosine, and tryptophan biosynthesis” pathways, the associated DAPs were mainly increased in abundance, whereas the corresponding DEGs were expressed in the opposite direction (Figure 4). After rehydration, there were eight associated DAPs (Supplementary Tables 5, 6). Unfortunately, some proteins were not annotated in the KEGG pathway database.

### Protein-protein interaction analysis

**FIGURE 5:**
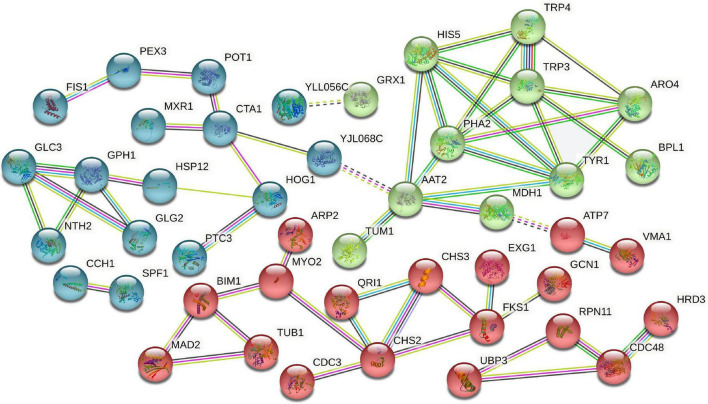
Protein-protein interaction analysis of dessication-responsive differentially abundant proteins. Different line colors represent the types of evidence used in predicting the associations: gene fusion (red), neighborhood (green), co-occurrence across genomes (blue), co-expression (black), experimental (purple), association in curated databases (light blue), or co-mentioned in PubMed abstracts (yellow).

Protein-protein interaction is essential for almost every process in cells and may be related to the specific function performed by proteins after binding into complexes through PPI. We selected 133 DAPs related to the dessication-stress response (e.g., stress response, sugar metabolism, and signal transduction) for PPI analysis. A total of 45 nodal DAPs were identified, which were divided into three clusters (Figure 5). Cluster 1 (blue bubbles) included 16 DAPs, which were mainly involved in stress response. CTA1 (catalase A), HOG1 (mitogen-activated protein kinase involved in osmoregulation), and PH1 (glycogen phosphorylase required for the mobilization of glycogen) were increased in abundance, which was conducive to the removal of excess reactive oxygen species, osmotic adjustment, and energy supply under dessication-stress.

Cluster 2 (green bubbles) included 11 more-abundant DAPs, most of which were related to amino acid metabolism. PHA2 (putative prephenate dehydratase) and TRP3 (multifunctional tryptophan biosynthesis protein) were important hub proteins in this cluster. The most nodal proteins were in the “phenylalanine, tyrosine, and tryptophan biosynthesis” and “tyrosine metabolism” pathways, and might participate in melanin synthesis.

Eighteen DAPs were found in cluster 3 (red bubbles), which were mainly involved in the synthesis and elasticity of the cell wall, such as chitin synthase II (CHS2), chitin synthase III (CHS3), and β-1,3-glucan synthase component (FKS1). These three kinds of proteins were less-abundant under dessication-stress and might contribute to dessication tolerance and the shrinkage of fruiting bodies.

### Important proteins associated with the response to dessication-stress

#### Melanin synthesis

Melanins are derived from tyrosine, and referred to as “fungal armor” due to the ability of the polymer to protect microorganisms against a broad range of toxic insults (Gómez and Nosanchuk, 2003; Glagoleva et al., 2020). Sixteen (12 more-abundant and four less-abundant) DAPs were identified in the “tyrosine metabolism” pathway (Table 1). The fold-change values of more-abundant proteins were higher than those that decreased. Tyrosine synthesis mainly exists in the “phenylalanine, tyrosine, and tryptophan biosynthesis” pathway. Fifteen DAPs were identified in this pathway, they were all increased in abundance. Among them, two proteins that directly promote tyrosine synthesis were histidinol-phosphate aminotransferase (Unigene7245_All, Log_2_FC = 2.60) and aspartate aminotransferase (CL2639.Contig2_All, Log_2_FC = 2.78) (Table 1). They provided a reactive substrate for melanin synthesis.

**TABLE 1:** Differentially abundant proteins in the “tyrosine metabolism” and “phenylalanine, tyrosine, and tryptophan biosynthesis” pathways.

Pathway	Protein ID	log_2_FC	Swissprot description	Gene name
Tyrosine metabolism	CL2936.Contig5_All	3.7326	–	–
	CL2639.Contig2_All	2.7795	Aspartate aminotransferase	*aat2*
	Unigene7245_All	2.6020	Histidinol-phosphate aminotransferase	*HIS5*
	CL1387.Contig4_All	2.5615	Copper amine oxidase 1	*cao1*
	Unigene219_All	2.3641	Tyrosinase-like protein	*orsC*
	CL2879.Contig1_All	2.3046	–	–
	CL6592.Contig1_All	2.2886	Uncharacterized oxidoreductase	*SPAC26H5.09c*
	CL5670.Contig1_All	1.7234	Alcohol dehydrogenase	*adh-1*
	CL1960.Contig4_All	1.7060	–	–
	CL1605.Contig3_All	1.5051	Uncharacterized oxidoreductase	*SPAC26H5.09c*
	CL1960.Contig3_All	1.3695	–	–
	CL6346.Contig2_All	1.3095	Dehydrogenase	*FUM7*
	Unigene76_All	−1.1781	UPF0303 protein	*YBR137W*
	CL1387.Contig2_All	−1.1973	Copper amine oxidase 1	*YBR137W*
	CL4484.Contig2_All	−1.4209	UPF0303 protein	*YBR137W*
	CL8627.Contig1_All	−1.6618	Tyrosinase-like protein	*orsC*
Phyenylalanine, tyrosine and tryptophan biosynthesis	CL4340.Contig1_All	3.2800	Multifunctional tryptophan biosynthesis protein	*TRPC*
	CL7709.Contig1_All	2.8417	Tryptophan synthase	*TRP-1*
	CL2639.Contig2_All	2.7795	Aspartate aminotransferase	*aat2*
	Unigene7245_All	2.6020	Histidinol-phosphate aminotransferase	*HIS5*
	CL2975.Contig3_All	2.4172	Probable prephenate dehydrogenase	*tyr1*
	CL8380.Contig2_All	2.2679	Putative prephenate dehydratase	*pha2*
	CL753.Contig4_All	2.1225	Phospho-2-dehydro-3-deoxyheptonate aldolase	*aro-8*
	Unigene3893_All	1.9892	–	–
	CL7603.Contig2_All	1.9430	Phospho-2-dehydro-3-deoxyheptonate aldolase	*aro4*
	CL2975.Contig2_All	1.8930	Probable prephenate dehydrogenase	*tyr1*
	CL3419.Contig1_All	1.7061	Probable anthranilate synthase component 1	*trp3*
	CL5368.Contig1_All	1.6306	Pentafunctional AROM polypeptide	*LACBIDRAFT_233717*
	CL702.Contig1_All	1.6001	–	–
	Unigene3701_All	1.0130	Chorismate synthase	*SPCC1223.14*
	CL1983.Contig2_All	1.0079	Anthranilate phosphoribosyltransferase	*trp4*

#### Vitamin synthesis

Tetrahydrofolic acid (THF) and its derivatives are known as folate or B9 vitamins (Basset et al., 2005). Biotin is also known as vitamin H or B7 (Lazar et al., 2017). The pathways of “folate biosynthesis” and “biotin metabolism” were remarkably enriched under dessication-stress (Figure 3C). In the “folate biosynthesis” pathway, 10 DAPs were identified, including six more-abundant and four less-abundant. Folylpolyglutamate synthase (CL112.Contig1_All) and probable dihydrofolate synthetase (CL8178.Contig1_All) were increased in abundance to promote folate synthesis and were consistent with the folate content (Figure 1D). Thirteen more-abundant DAPs were identified in the “biotin metabolism” pathway (Table 2). Among them, the fold-change value of CL4671.Contig1_All was the highest (4.51) (Table 2). This was consistent with the biotin content (Figure 1B).

**TABLE 2:** Differentially abundant proteins in “folate biosynthesis” and “biotin metabolism” pathways.

Pathway	Protein ID	log_2_FC	Swissprot description	Gene name
Folate biosynthesis	CL3176.Contig2_All	3.6059	Short chain dehydrogenase	*yanD*
	CL112.Contig1_All	2.8412	Folylpolyglutamate synthase	*MET7*
	CL7813.Contig2_All	2.0205	–	–
	CL4756.Contig1_All	1.7722	Uracil-regulated protein 1	*urg1*
	CL7253.Contig3_All	1.2942	–	–
	CL8178.Contig1_All	1.0659	Probable dihydrofolate synthetase	*fol3*
	Unigene10797_All	−1.3183	–	–
	CL5565.Contig2_All	−1.7700	Uncharacterized protein	*SPAC57A10.07*
	CL1254.Contig1_All	−1.9403	Repressible alkaline phosphatase	*PHO8*
	CL1254.Contig2_All	−2.1234	Repressible alkaline phosphatase	*PHO8*
Biotin metabolism	CL4671.Contig1_All	4.5140	Uncharacterized oxidoreductase	*SPBC30D10.05c*
	CL1463.Contig1_All	3.9789	Uncharacterized oxidoreductase	*SPAC4H3.08*
	CL3919.Contig4_All	2.9139	Uncharacterized oxidoreductase	*SPBC30D10.05c*
	Unigene347_All	2.7646	Uncharacterized oxidoreductase	*SPCC663.06c*
	CL8130.Contig2_All	2.6830	Biotin–protein ligase	*bpl1*
	CL1463.Contig2_All	2.6794	Uncharacterized oxidoreductase	*SPAC4H3.08*
	CL2219.Contig1_All	2.4481	Short chain dehydrogenase	*citE*
	CL5550.Contig1_All	1.9873	Probable NADP-dependent mannitol dehydrogenase	*YALI0B16192g*
	CL3784.Contig3_All	1.6988	Versicolorin reductase	*stcU*
	CL5385.Contig4_All	1.6626	Uncharacterized oxidoreductase	*SPAC4H3.08*
	CL4968.Contig1_All	1.5692	Trihydroxynaphthalene reductase	*THR1*
	CL3093.Contig2_All	1.4250	Uncharacterized oxidoreductase	*SPAC4H3.08*
	CL8130.Contig3_All	1.4111	Biotin–protein ligase	*bpl1*

#### Antibiotic synthesis

**FIGURE 6:**
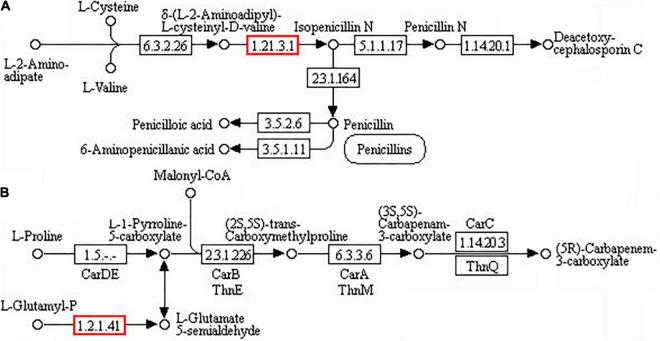
“Biosynthesis of antibiotics” pathway. **(A)** Biosynthesis of penicillin and cephalosporin. **(B)** Carbapenem biosynthesis.

The associated number of DAPs/DEGs in the “biosynthesis of antibiotics” pathway was the greatest under dessication-stress by proteome–transcriptome-associated analysis. There were 135 DAPs enriched in this pathway, of which 117 DAPs were increased in abundance, and the fold-change value of more-abundant DAPs was much higher than that of the less-abundant value. The top 12 DAPs with | log_2_FC| ≥ 3 were increased in abundance (Table 3). Two DAPs (CL711.Contig2_All and CL6373.Contig2_All) were involved in isopenicillin-N synthase and glutamate-5-semi aldehyde dehydrogenase, respectively, and could enhance the synthesis of penicillin, cephalosporins, or carbapenem (Figure 6). Other 132 DAPs could promote intermediate synthesis of penicillin, carbapenem, or cephalosporin. For example, fumarase (CL6387.Contig1_All) could catalyze the conversion of fumarate to malate (Nunes-Nesi et al., 2007), then malate is oxidized to oxoloacetate by NAD^+^-dependent malate dehydrogenase (Nunes-Nesi, 2005). Oxoloacetate facilitates the synthesis of cysteine, and L-cysteine is the precursor for penicillin and cephalosporin synthesis in the “penicillin and cephalosporin biosynthesis” pathway.

**TABLE 3:** Highly differentially abundant proteins in the “biosynthesis of antibiotic” pathway.

Protein ID	log_2_FC	Swissprot description
CL4671.Contig1_All	4.5140	Uncharacterized oxidoreductase
CL1006.Contig5_All	4.3139	AB hydrolase superfamily protein
CL1463.Contig1_All	3.9789	Uncharacterized oxidoreductase
CL5160.Contig2_All	3.7181	3-ketoacyl-CoA thiolase
CL3888.Contig1_All	3.5051	Uncharacterized protein
CL1870.Contig3_All	3.4639	Phosphoglucomutase
Unigene388_All	3.4583	Branched-chain-amino-acid aminotransferase
Unigene385_All	3.4537	3-ketoacyl-CoA thiolase
CL1155.Contig1_All	3.4053	Probable aldose 1-epimerase
CL4340.Contig1_All	3.2800	Multifunctional tryptophan biosynthesis protein
CL6387.Contig1_All	3.1756	Fumarate hydratase
Unigene17153_All	3.0785	Uncharacterized oxidoreductase

#### Cytoskeleton

**FIGURE 7:**
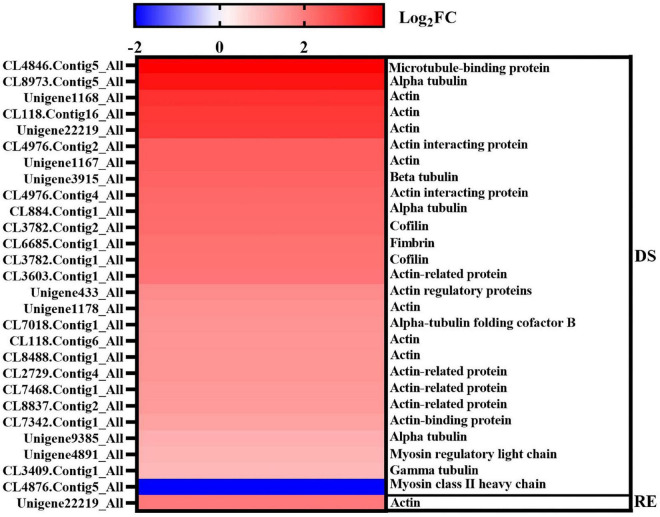
Differentially abundant proteins related to the cytoskeleton under dessication and rehydration. The left and right Y axes represent the protein ID and functional annotation, and DS and RE represent dessication-stress and rehydration, respectively.

The fruiting bodies of *A*. *fibrillifera* shrank and became hard under dessication-stress, whereas they recovered rapidly after rehydration (Figure 1A). Long-lived cytoskeleton structure may be an epigenetic regulator of cellular function and fate (Fletcher and Mullins, 2010). A total of 28 (27 more-abundant and one less-abundant) DAPs were associated with cytoskeleton under dessication and rehydration conditions, and the fold-change of more-abundant proteins was much higher than that of less-abundant proteins (Figure 7). These DAPs usually had high fold-change, among which the | log_2_FC| value of 16 proteins was ≥ 2. CL4846.Contig5_All (Log_2_FC = 3.87) and CL8973.Contig5_All (Log_2_FC = 3.55) had the highest fold differences, which play roles in microtubule-related proteins and tubulin alpha chain, respectively (Figure 7). The functions of other significantly more-abundant DAPs were mainly concentrated in actin, tubulin, fimbrin, and cofilin. Fimbrin and cofilin bind to actin to function. The number of DAPs decreased rapidly after rehydration. The DAP (Unigene22219_All) with the highest fold-change (Log_2_FC = 2.02) was also related to actin (Figure 7).

## Discussion

Proteomics technology is a tool for the comprehensive identification of plant proteins related to drought resistance (Gupta et al., 2020; Qiu et al., 2021). Proteome research has been successfully applied in drought-resistant crops, such as cucumber (Du et al., 2019), corn (Liu et al., 2019), and cassava (Zhao et al., 2015). The strategies of plants to deal with drought stress usually include osmotic regulation, antioxidant capacity, and dehydration tolerance (Zhang, 2007). Similar results were obtained in this experiment. In PPI cluster 1, the DAPs with stronger interactions were concentrated mainly in the above functions. In addition, the DAPs with high fold-change, such as CL4047.Contig1_All (Log_2_FC = 5.02), CL1516.Contig1_All (Log_2_FC = 4.93), CL7279.Contig1_All (Log_2_FC = 4.57), and Unigene10667_All (Log_2_FC = 4.50) were mainly related to antioxidant mechanisms and heat shock proteins in response to dessication-stress. Meanwhile, the excellent dessication tolerance of *A*. *fibrillifera* might be caused by secondary metabolites (melanin, antibiotics, and vitamins).

### Melanin response to dessication-stress

Melanin protects dark-pigmented fungi from environmental stresses (Brunskole et al., 2009; Li et al., 2019). Plants highly pigmented are more resistant to biotic and abiotic stresses (Carletti et al., 2014). Melanin has a variety of functions, structures, and forms, which can resist a variety of abiotic factors (Cordero and Casadevall, 2020). Melanin compounds are endowed with excellent photoprotective properties and antioxidative activity (Liberti et al., 2020). Melanin has both free radical scavenging and antioxidant activities. The melanin of *Aureobasidium melanogenum* XJ5-1 in the Taklimakan Desert plays a vital role in the adaptation of yeasts to drought stress (Jiang et al., 2018). Here, the “phenylalanine, tyrosine and tryptophan biosynthesis” pathway was significantly enriched. TRP3 in this pathway played a central role and had the strongest interaction with other proteins in the PPI analysis. Physiological data showed that the level of melanin increased more than 10-fold under dessication-stress. Tyrosine is the precursor of melanin formation (Gómez and Nosanchuk, 2003; Micillo et al., 2016). In the “tyrosine metabolism” pathway, the melanin-related protein Unigene219_All (Log_2_FC = 2.36) was significantly increased in abundance too. Under dessication-stress, the DAPs involved in melanin synthesis were significantly increased in abundance, and the melanin content was significantly increased. Proteome data were consistent with the above melanin content. Therefore, specific melanin should contribute to high desiccation tolerance because of its antioxidant and free-radical-scavenging activities in *A*. *fibrillifera*.

### Vitamin response to dessication-stress

Folate plays a crucial role in overcoming drought-stress during plant development. The foliar application of folate was found to be suitable for drought stress alleviation in *Coriandrum sativum* L. (Khan et al., 2022). In addition, folates are necessary for the biosynthesis of lignin (Hanson and Gregory, 2011). In this experiment, “folate biosynthesis” and “biotin metabolism” pathways were significantly enriched under dessication-stress. The “folate biosynthesis” pathway was enriched by both transcriptomic and proteomic analysis. The DAP CL112.Contig1_All in this pathway played a role in folylpolyglutamate synthase. This enzyme was very important for maintaining folate homeostasis (Strobbe and Van Der Straeten, 2017). The folate content increased significantly under dessication-stress, whereas it decreased significantly after rehydration. Therefore, folate was the most sensitive to both drought stress and rehydration, and may have a close relation to drought tolerance in *A*. *fibrillifera*.

Biotin not only plays a key role in immune regulation in animals but also in the response of plants to various abiotic stresses. Biotin enhances the resistance of *Arabidopsis* to carbonate stress (Wang et al., 2020). Biotin synthetases and biotin attachment domain containing protein were identified in the root tip of soybean, indicating that biotin and biotinylation were involved in glucose metabolism under drought stress (Wang et al., 2016). In this work, the DAPs and their associated DEGs were up-regulated in the “biotin metabolic” pathway. The significant abundance increase of biotin-related DAPs might supply energy sources to *A*. *fibrillifera* under dessication-stress.

### Antibiotics involved in dessication tolerance

Antibiotics are a group of secondary metabolites generated by microorganisms or higher organisms in life processes (Mohr, 2016). “Biosynthesis of antibiotics” pathways were significantly enriched in six wheat genotypes under drought stress (Rasool et al., 2022). The enhancement of pyrimidine nucleoside antibiotics can alleviate abiotic stress in *Nicotiana tabacum* (Song et al., 2022). Penicillins and cephalosporins are the most important classes of β-lactam antibiotics. In this experiment, antibiotic-related DAPs were highly increased in abundance, including fumarate hydratase (FH) (CL6387.Contig1_All), isopenicillin-N synthase (IPNS) (CL711.Contig2_All), and glutamate-5-semialdehyde dehydrogenase (CL6373.Contig2_All), which are beneficial to the synthesis of β-lactam antibiotics. FH facilitates the synthesis of cysteine, which is the precursor for penicillin and cephalosporin synthesis. IPNS can catalyze the specific reaction of L-δ-(α-aminoadipoyl)-L-cysteinyl-D-valine with dioxygen giving isopenicillin-N, a precursor of cephalosporins and penicillins (Rabe et al., 2021). The significant abundance increase of these DAPs under dessication-stress is beneficial to the accumulation of penicillin and cephalosporin in *A*. *fibrillifera*. In the “carbapenem biosynthesis” pathway, glutamate-5-semialdehyde dehydrogenase-related DAP was significantly increased in abundance and might be beneficial to the synthesis of carbapenem. In transcriptome-proteome-association analysis, many DEGs/DAPs were enriched in the “biosynthesis of antibiotics” pathway, which could be beneficial to dessication tolerance in *A*. *fibrillifera*.

### Cytoskeleton response to dessication-stress

The cytoskeleton is the main mechanical structure of cells; it is a complex and dynamic biopolymer network composed of microtubules, actin, and intermediate filaments (Pegoraro et al., 2017). The plant cytoskeleton is associated with plant stress responses, such as drought, salt, and cold (Chun et al., 2021). A balance in the metabolism of cell wall component biosynthesis and cytoskeleton homeostasis can affect the response of cotton fibers to drought stress (Zheng et al., 2014). The microtubule cytoskeleton functions as a sensor for stress response signaling in plants and maintains mechanical stability by forming bundles (Ma and Liu, 2019). Furthermore, actin filaments may control drought-induced signal perception and are involved in regulating the accumulation of *HVA1* (a dehydrin-encoding gene) transcripts in barley leaves exposed to drought stress (Śniegowska-Świerk et al., 2016). In this experiment, the fruiting bodies of *A*. *fibrillifera* shrink under dessication-stress. A total of 28 DAPs were associated with the cytoskeleton under dessication-stress and rehydration conditions, and the fold-change of more-abundant proteins was much higher than that of less-abundant proteins (Figure 7). The functions of significantly more-abundant DAPs were mainly concentrated in actin, tubulin, fimbrin, and cofilin. Fimbrin and cofilin bind to actin to function. The number of DAPs decreased rapidly after rehydration. The DAP (Unigene22219_All) with the highest fold-change was also related to actin. After rehydration, the fruiting bodies could quickly absorb water and return to the control level, which might be related to the rapid assembly or disassembly of actin. Therefore, the DAPs related to the cytoskeleton might be helpful in adaptation to dessication tolerance and the shape changes of fruiting bodies.

## Conclusion

The levels of melanin, amino acids, folate, and biotin in fruiting bodies increased significantly under dessication-stress compared with those of the controls. Folate showed a sensitive response to both dessication-stress and rehydration. In total, 1,572 DAPs were identified under dessication-stress. The number of DAPs decreased rapidly after rehydration. Under dessication-stress, many DAPs were annotated in “amino acid metabolism,” “carbohydrate metabolism,” “folate biosynthesis,” “biotin metabolism,” “biosynthesis of antibiotics,” and “transport and catabolism” pathways. Proteome–transcriptome association analysis revealed that “biosynthesis of antibiotics,” “folate biosynthesis,” “biotin metabolism,” “phenylalanine, tyrosine, and tryptophan biosynthesis” pathways were significantly enriched in response to dessication-stress. The DAPs in these pathways were beneficial to the synthesis of antibiotics, folate, biotin, and melanin, and played important roles in enhancing the dessication tolerance of *A*. *fibrillifera*. The findings of transcriptome and physiological analyses were in good agreement with the proteomic data. Some molecular pathways and mechanisms of dessication response are similar between *A*. *fibrillifera* and plant species. This work may shed light on the mechanism of dessication tolerance and provide a novel framework for the breeding and cultivation of *Auricularia* and crops.

## Data availability statement

The data presented in this study are deposited in the ProteomeXchange repository, accession number PXD033449.

## Author contributions

SZ and GG designed the experiments. HG, XX, YW, and HT were performed material preparation, data collection, and analysis. HG, XX, YW, HT, SZ, and GG wrote the manuscript. All authors have read and approved the final manuscript.

## Supplementary material

Supplementary Figure 1 Heatmap of sample correlation analysis. Both X and Y axes represent samples. The color denotes the correlation coefficient. CK1, CK2, DS, and RE represent the parallel control of desiccation-stress, parallel control of rehydration, desiccation-stress, and rehydration process, respectively. “−1,” “−2,” and “−3” indicate three biological replicates.
