# Supplementary figures and images for: Integrative proteomic and physiological analyses of the molecular response to dessication-stress in Auricularia fibrillifera
Source: Front Plant Sci. 2022 Sep 21;13:995810. doi: 10.3389/fpls.2022.995810 (PMC9532602; doi:10.3389/fpls.2022.995810)

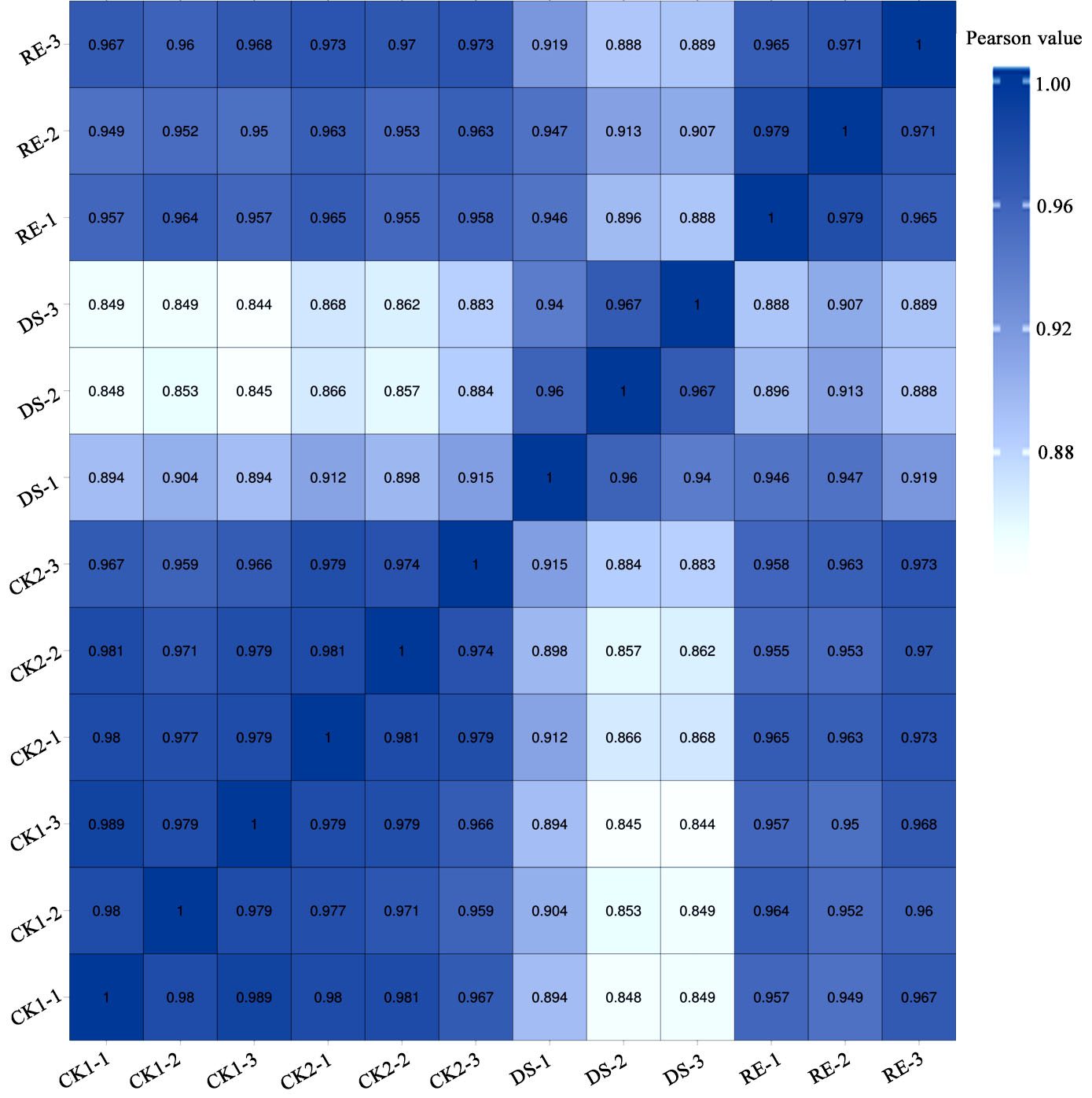

Supplement: Supplementary Figure 1 — Heatmap of sample correlation analysis. Both X and Y axes represent samples. The color denotes the correlation coefficient. CK1, CK2, DS, and RE represent the parallel control of desiccation-stress, parallel control of rehydration, desiccation-stress, and rehydration process, respectively. “−1,” “−2,” and “−3” indicate three biological replicates. [file Image_1.JPEG]
